# Macrophages and angiogenesis: a role for Wnt signaling

**DOI:** 10.1186/2045-824X-4-13

**Published:** 2012-08-31

**Authors:** Andrew C Newman, Christopher C W Hughes

**Affiliations:** 1 The Department of Molecular Biology and Biochemistry, University of California Irvine, Irvine, CA, 92697, USA; 2 The Department of Biomedical Engineering, University of California Irvine, Irvine, CA, 92697, USA; 3 The Edwards Lifesciences Center for Advanced Cardiovascular Technology, University of California Irvine, Irvine, CA, 92697, USA

**Keywords:** Macrophage, Angiogenesis, Wnt

## Abstract

Macrophages regulate many developmental and pathological processes in both embryonic and adult tissues, and recent studies have shown a significant role in angiogenesis. Similarly, Wnt signaling is fundamental to tissue morphogenesis and also has a role in vascular development. In this review, we summarize recent advances in the field of macrophage-regulated angiogenesis, with a focus on the role of macrophage-derived Wnt ligands. We review data that provide both direct and indirect evidence for macrophage-derived Wnt regulation of physiologic and pathologic angiogenesis. Finally, we propose that Wnt signaling plays a central role in differentiation of tumor associated and wound infiltrating macrophages to a proangiogenic phenotype.

## Introduction

Angiogenesis is the growth of new blood vessels from the existing vasculature. This process plays critical roles in both development and pathological conditions such as tumor growth and diabetic retinopathy [1]. During angiogenesis, endothelial cells (EC) coordinate signals derived from a multitude of stromal cells in close proximity to the blood vessels, including fibroblasts, smooth muscle cells/pericytes and macrophages [2]. Growth factors, proteases and extracellular matrix (ECM) components derived from these stromal cells participate in the process [1]. In this review, we will focus on the role of macrophage-derived Wnt signals in regulating angiogenesis.

Members of the Wnt protein family have been shown to regulate diverse biological processes including cell proliferation, apoptosis, polarity, differentiation and the maintenance of pluripotency in stem cells [3]. Wnt proteins have been studied extensively, but only recently has their function in angiogenesis begun to be elucidated. Macrophages comprise a diverse group of cells from the mononuclear phagocytic lineage [4]. They are highly plastic, exhibiting dramatic changes in phenotype in response to various stimuli and, in addition, are believed to exist in a multitude of subpopulations [4-6]. This review is not intended to be an overview of different macrophage phenotypes and subpopulations, instead, we highlight studies showing an angiogenic function for a variety of the different subpopulations of macrophages. We will employ a general definition of macrophages as any non-dendritic cell of monoblast origin, which includes monocytes and the specialized tissue-resident macrophages of the central nervous system, microglial cells. Increasing evidence suggests that macrophages mediate their effects on angiogenesis, in part, through secretion of members of the Wnt family of secreted glycoproteins.

## The role of Wnts in angiogenesis

In humans, Wnt signaling is mediated by a family of 19 secreted Wnt glycoproteins and 10 transmembrane Frizzled (Fzd) receptors [7]. Although the distinction is becoming less clear [8,9] Wnt signaling is generally broken down into the canonical, Wnt/β-catenin pathway, and the non-canonical, Wnt/Calcium and planar cell polarity (PCP) pathway. In the canonical Wnt pathway, Wnt ligand binding to Fzd and the co-receptors, LDL-receptor-related proteins-5 or −6 (Lrp5/6), leads to cytosolic β-catenin accumulation. β-catenin then translocates to the nucleus where it binds to transcription factors of the lymphocyte enhancing factor (LEF)/T-cell factor (TCF) family, thereby activating transcription of a number of target genes [7,10]. Non-canonical Wnts signal through Fzd receptors as well as members of the receptor tyrosine kinase-like orphan receptor (Ror) family and the Wnt modifier, receptor-like tyrosine kinase (Ryk). This pathway leads to changes in cell polarity and migration and is mediated by Ca^2+^influx as well as activation of the small GTPases, RhoA, Cdc42 and Rac [11,12]. A detailed overview of Wnt signaling is beyond the scope of this review and the reader is referred to many excellent reviews [7,8,10,11] as well as the Wnt Gene Homepage at http://www.stanford.edu/~rnusse/wntwindow.html.

Evidence for Wnt signals regulating angiogenesis is provided firstly by reports of ECs expressing a number of Wnt receptors including Fzd1, Fzd2, Fzd4, Fzd5, Fzd6, Fzd7, Fzd9, Fzd10, Lrp5, Lrp6 and the Wnt signaling modulator,Ryk [13-16]. It is not surprising then, that there are numerous reports showing that EC respond to Wnt proteins in vitro. Wnt1, Wnt3a and Wnt5a have all been shown to control EC proliferation and in some cases migration [17-23], two processes critical for angiogenesis. In addition, the Wnt antagonist, secreted frizzled-related protein 1 (sFRP1) reduces EC proliferation [24] and canonical Wnt signaling was shown to promote tube-like formation on Matrigel [25]. Masckauchan et al. have shown that Wnt5a induces expression of Tie-2 in human umbilical vein endothelial cells (HUVEC), a receptor tyrosine kinase involved in EC survival and maturation [19] and we have independently confirmed this finding in our lab (unpublished observations). Thus, it is clear that Wnt signaling pathways can regulate angiogenesis in vitro.

The story is much the same in vivo, with several reports of active Wnt signaling in EC undergoing angiogenesis [26-33]. Moreover, many Wnt signaling pathway alterations lead to vascular defects. β-catenin nuclear localization has been observed in capillaries during human development [28] and various reporter mice have shown that canonical Wnt signaling is active in EC during developmental angiogenesis [27,30,31]. β-catenin has also been shown to accumulate in the nucleus of EC undergoing pathological angiogenesis in rat models of myocardial infarction [26] and glioma [32] as well as in human tumors of the central nervous system [29,33]. Of note, β-catenin nuclear localization is rarely seen in adult quiescent EC [26,29,32,33]. Changes in Wnt signaling pathways have also been correlated with angiogenesis. For example, endothelial-specific β-catenin gain-of-function mutant mice are embryonic lethal and display decreased vessel lengths and increased lumen diameter [34]. These mice also display upregulated Notch signaling, another important mediator of vessel growth [34]. Injection of cells overexpressing Wnt1 and Wnt3a into the chick paraxial mesoderm leads to increased vascular density [35], and disruption of the Wnt2 gene in mice results in placental vascular defects with a decrease in the number of fetal capillaries [36].

Disturbing Wnt receptor expression in vivo can also lead to vascular defects, with the most well-studied receptors being Fzd4 and Fzd5. Fzd4 loss-of-function mutations have been linked to familial exudative vitreoretinopathy (FEVR), a disease characterized by abnormal retinal vasculature [37]. Furthermore, Fzd4 deletion in mice has been shown to alter vessel formation in the cerebellum and the retina [38]. However, at least in the retina, one Fzd4 ligand is Norrin, which is not a Wnt family member, but does activate the canonical Wnt signaling pathway [38]. In a recent study, Descamps et al. showed that Fzd4^−/−^ mice display significant reductions in arteriole density in the heart and kidney as well as impaired angiogenesis in a mouse model of hind limb ischemia [39]. The evidence presented in that study suggests that the Fzd4 ligand is a non-canonical Wnt. Fzd5 knockout (KO) mice are embryonic lethal due to defects in yolk sac angiogenesis. Specifically, EC proliferation is impaired and an incomplete capillary plexus is formed [40]. A more recent study using a conditional genetic deletion of Fzd5 to bypass the perinatal lethality observed in the original KO mouse, shows that this Wnt receptor also regulates blood vessel development [41].

Clearly there is a plethora of evidence implicating Wnt signaling in modulating angiogenesis. For a more extensive overview of the literature, the reader is referred to several recent reviews [42-44].

## Macrophages in angiogenesis

There is substantial evidence that macrophages play a significant role in both physiological and pathological angiogenesis [4,45,46]. In mouse development the first macrophages appear at 7.5 days post-coitum (dpc) and starting between 8 and 9.5 dpc they can be found in both extra-embryonic and embryonic tissue [47], placing them in prime position to regulate angiogenesis. In support of this idea, embryonic macrophages, compared to circulating adult macrophages, have been shown to have “wound healing” and “angiogenesis” gene expression signatures [48]. Macrophages colonize the mouse embryonic hindbrain independently of blood vessel formation and Fantin et al. showed that these tissue-resident macrophages associated with angiogenic tip cells during the same time period that neighboring vessels were anastomosing with one another [49]. Ablation of macrophages, using both PU.1^−/−^ and Csf-1^op/op^ mice, resulted in a decreased number of vessel intersections in the hindbrain, providing the first evidence that macrophages actively participate in vessel anastomosis [49]. Interestingly, these tissue-resident macrophages were shown to express Tie-2 and NRP1, two genes that are known to be significantly upregulated in proangiogenic macrophages [47]. Also of note, the effect of these macrophages on angiogenesis is not mediated by vascular endothelial growth factor-A (VEGF-A) secretion [49]. However, the study does not rule out the possibility that macrophages secrete other VEGF isoforms that contribute to angiogenesis. Evidence for macrophages regulating vessel anastomosis in other tissues comes from studies in the mouse retina [49-51]. Outtz and colleagues provided evidence that macrophage Notch1 regulates this process [50] and studies from Rymo et al. indicate that microglial-secreted factors other than VEGF-A increase vessel sprouting and branching in the rat aortic ring model of angiogenesis [51].

Macrophages, given their ability to migrate within virtually all tissues of the body, are also an ideal cell to regulate angiogenesis during tissue injury and repair. Using, a transgenic mouse model of ischemic cardiomyopathy, Moldovan et al. showed that macrophages carved out tunnels in the ECM, thereby providing avenues for subsequent capillary infiltration [52]. Macrophages are actively recruited to wound sites and macrophage-depletion during early phases of the wound healing process leads to reduced formation of vascularized granulation tissue, whereas depletion during later phases caused severe hemorrhage and prevented wound closure [53]. These findings implicate macrophages in mediating both the initial and maturation stages of angiogenesis. A separate study showed that wound healing was delayed in macrophage-deficient Csf-1^op/op^ mice and this correlated with decreased vascular density [54]. Thus, although it is well known that macrophages serve an immune function during tissue repair, it is also clear that they play a trophic role through the regulation of angiogenesis.

A rapidly developing field is the study of tumor-associated macrophages (TAMs) and their role in promoting tumor progression. It comes as no surprise that TAMs mediate some of their effects on tumor growth by regulating angiogenesis [45,46,55,56]. TAMs can be recruited to tumors by a number of different cytokines, including CCL2 and CSF-1/M-CSF [57-59]. TAMs have been shown to regulate tumor angiogenesis in a number of different tissues including bone, brain, breast, cervix, colon and lung, among others [60-66]. They have been shown to mediate angiogenesis in part by secretion of VEGF, along with proteases, such as matrix metalloproteinases (MMPs) [64,67]. Interestingly, studies by De Palma and colleagues have shown that the majority of proangiogenic TAMs in tumors are Tie-2 expressing monocytes (TEMs) [47,68], implicating Tie-2 as an important marker of proangiogenic macrophages. Indeed, Angiopoietin-2 (Ang-2) signaling via Tie-2 in TEMs has been shown to upregulate proangiogenic genes [69]. Although TEMs have been shown to exist as a subset of circulating monocytes [68], studies have shown that conditions of hypoxia can upregulate Tie-2 expression in monocytes [70]. As in EC [19], Tie-2 expression in macrophages may also be regulated by Wnt5a. It is clear that macrophages regulate angiogenesis through multiple mechanisms, however, a detailed discussion on the role of macrophage-derived cytokines in angiogenesis is beyond the scope of this review.

## Macrophage-derived Wnts regulate angiogenesis

Based on the studies outlined above, it is evident that both macrophages and secreted Wnt proteins regulate angiogenesis. It may, therefore, be reasonable to hypothesize that Wnt ligands mediate some of the effects that macrophages have on angiogenesis, and indeed, macrophages do express Wnt ligands. Interferon-γ (IFN-γ) and lipopolysaccharide (LPS), two potent inducers of inflammation, lead to significant upregulation of Wnt5a transcript and protein levels in macrophages [71]. Furthermore, Wnt5a can signal in an autocrine manner to induce expression of several pro-inflammatory cytokines in macrophages including IL-6, IL-8 and IL-1β [71], all of which have been shown to be pro-angiogenic [72-75]. Given that Wnt5a has also been shown to induce expression of several inflammatory cytokines in EC, including IL-6 and IL-8 [76]combined with the direct effects that Wnt5a has on EC proliferation, migration and Tie-2 expression [18,19,22], it is reasonable to hypothesize that macrophage-derived Wnt5a regulates angiogenesis via both direct and indirect mechanisms and at multiple levels. Moreover, as Wnt5a has also been shown to upregulate expression of the macrophage chemotactic protein, CCL2, in EC [76], it is possible that macrophage-derived Wnt5a could indirectly drive additional rounds of macrophage recruitment.

Aside from inflammatory angiogenesis, research in cancer biology also points to a role for macrophage-derived Wnts in regulating tumor angiogenesis. Smith et al. looked at Wnt expression in 14 matched cases of normal, adenomatous and malignant colorectal tissues and reported upregulation of Wnt gene expression in TAMs, in particular, Wnt2 and Wnt5a, during the progression from normal through adenoma to carcinoma in colorectal tissue [77]. Of note, the angiogenic switch in colorectal cancer is thought to occur during the progression from adenoma to carcinoma [78]. As mentioned earlier, TAMs play an essential role in breast cancer angiogenesis [60,61]. Ojalvo et al. separated “invasive” TAMs from general TAMs using an in vivo migration assay [79] combined with fluorescent activated cell sorting (FACS) [80], and used gene expression analysis to show that “invasive” TAMs were enriched for Wnt signaling molecules [80]. Based on these findings and others, Qian and Pollard recently hypothesized that this subset of Wnt-expressing-TAMs may link angiogenesis and tumor invasion [46]. Further investigation is needed to confirm the role of macrophage-derived Wnts in regulating colorectal and breast tumor angiogenesis.

Direct evidence for the involvement of macrophage-derived Wnts in regulating hyaloid vascular remodeling and developmental angiogenesis in the retina comes from studies by Lang and colleagues. The hyaloid vessel system is laid down during development of the eye and requires remodeling after birth to allow for unobstructed vision [81]. Macrophages are in close contact with these vessels and genetic approaches that deplete macrophages lead to a loss of remodeling and the persistence of these vessels post-natally [82]. Further examination revealed that macrophage secretion of the canonical Wnt protein, Wnt7b, is required for this process [83]. Specifically, pericyte secretion of Ang-2 induces Wnt7b expression in macrophages; Wnt7b then stimulates vascular EC entry into the S phase of the cell cycle. Pericyte secretion of Ang-2 inhibits Ang-1-mediated survival signaling in EC and also signals through β-catenin to induce cell cycle entry and subsequent apoptosis [83,84]. The authors hypothesize that the need for macrophage-derived Wnt7b allows for coordination of apoptosis and phagocytosis [84].

Non-canonical Wnts secreted by macrophages also regulate vessel branching in the mouse retina. Shortly after birth, vessels from the superficial vascular plexus on the surface of the retina sprout vertically, down through the ganglion. Upon reaching the outer edge of the inner nuclear layer, EC turn and branch to form the deep vascular plexus [85]. Stefater et al. showed that retinal myeloid cells (RMC) are in close contact with tip cells of EC sprouts at the point of branching and remained associated after EC extend within the plane of the deep retinal layer [86]. They further showed that this population of RMCs is different than RMCs found in the superficial vascular layer. Specifically, they express the non-canonical Wnt ligands, Wnt5a and Wnt11, whereas superficial RMCs do not [86]. The authors also demonstrated that myeloid-specific deletion of the gene for the Wnt ligand transporter Wls, a protein required for Wnt secretion [87], resulted in increased vascular density of the deep vascular plexus, a phenotype shared by Wnt5a^+/−^ and Wnt11^+/−^ heterozygotes. The mechanism was shown to involve an autocrine-signaling axis where macrophage-derived Wnt5a and Wnt11 induce secretion of the soluble VEGF inhibitor Flt1 [88,89] that, in turn, inhibits vessel branching [86].

It is clear that Wnt signaling is important for angiogenic regulation by macrophages and we anticipate that future research in this field will reveal an even more crucial role for these proteins in this process.

## Conclusions and perspective

We have laid out evidence indicating that macrophages regulate angiogenesis, in part, through secreting and responding to Wnt signaling glycoproteins. This is true in both developmental and pathological angiogenesis. It is interesting and perhaps not unexpected that in some cases Wnts seem to be pro-angiogenic, as is the case for Wnt5a-induced EC proliferation and migration [19,20], and in other cases inhibitory, for example Wnt5a inhibition of vessel branching in the retinal deep vascular plexus [86]. One possible explanation for this relates to the fact that angiogenesis is a *series* of events with different morphological changes required at different stages. Therefore, the same protein may inhibit or induce angiogenesis depending on the stage of angiogenesis at which it is present. We also reason that these differences are due to differential Wnt receptor expression on the various cells responding to the signal as well as different signaling proteins that are present in the microenvironment. Coordinated crosstalk of Wnt signaling pathways with other pathways in EC such as Notch/Dll4, VEGF, Ang1/2 and focal adhesion kinase (FAK) [90] ultimately will determine the nature of the response. Furthermore, the cell population in a given tissue will likely alter the angiogenic response to macrophage-derived Wnts. We have discussed Wnt signaling in ECs and macrophages, but it is likely that Wnt binding to fibroblasts, smooth muscle cells and pericytes alters their phenotype as well. In the cases described above, differences in the effect of Wnt5a on angiogenesis could be explained by which cells bind the Wnt5a ligand. Wnt5a binding directly on the EC induces proliferation and migration, whereas Wnt5a signaling in an autocrine manner in macrophages leads to upregulation of the soluble VEGF inhibitor, Flt1, to reduce vessel branching.

**Figure 1:**
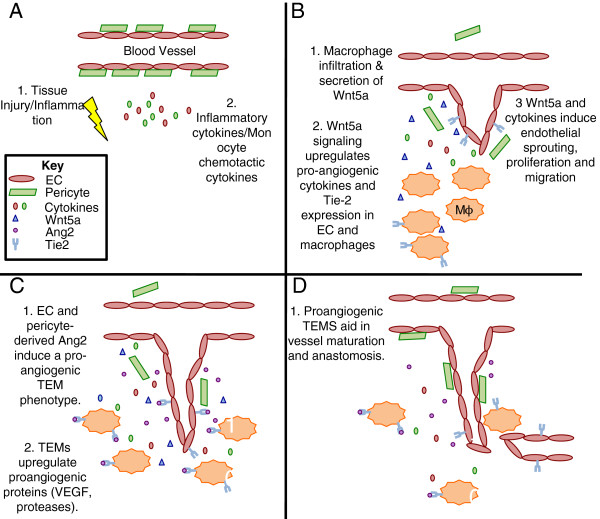
**Model for macrophage-derived Wnt5a regulation of angiogenesis.(A)** A wound or inflammation induces secretion of cytokines, including the macrophage chemotactic cytokines M-CSF and CCL2, by fibroblasts, pericytes, EC and other stromal cells present at the site of injury. **(B)** Monocytes extravasate from the blood vessels and secrete Wnt5a, which upregulates expression of Tie2 in macrophages and EC (note: basal levels of Tie-2 expression not depicted) as well as induces proliferation and migration of sprouting EC. **(C)** EC and pericyte-derived Ang2 signals through Tie2 expressed on the surface of TEMs, further polarizing macrophages to a proangiogenic phenotype. **(D)** TEMs then participate in the maturation phases of angiogenesis, including vessel lumen formation and anastomosis.

Based on the studies that we have presented in this review, we hypothesize that Wnt5a is a crucial mediator of macrophage phenotype. It is known that Wnt5a induces Tie-2 expression in EC [19] and it would be interesting to determine if macrophages respond in the same manner, especially as TEMs are known to be critical mediators of angiogenesis [47,68,69]. This would implicate Wnt5a as an important determinant of macrophage differentiation from an inflammatory function to a more trophic function. We propose a model where wounding or inflammation leads to a release of inflammatory cytokines, including the macrophage chemotactic proteins CCL2 and M-CSF (Figure 1A). Upon recruitment, macrophages release Wnt5a, which induces EC proliferation and migration, but also upregulates Tie-2 expression in macrophages and EC (Figure 1B). These Tie-2 expressing macrophages (TEM) respond to Ang-2 secreted by EC and pericytes and polarize into a more proangiogenic macrophage [69] (Figure 1C). During later stages of angiogenesis these TEMs aid in vessel anastomosis and maturation (Figure 1D).

Additionally, studies of TAMs show that “invasive” TAMs in breast cancer are enriched for Wnt signaling pathway components [80] and colon cancer progression correlates with increased Wnt5a expression in TAMs [77]. These observations, combined with studies implicating TEMs as the predominant proangiogenic macrophage within the tumor microenvironment [47,68], suggest that this model may be relevant to tumor angiogenesis as well, especially in light of the tumor being a “wound that does not heal” [91]. Owing to the perinatal lethality exhibited by Wnt5a-null mice [92], it has been difficult to study its function in vivo. Until conditional Wnt5a KO mice are made, we anticipate that complex three-dimensional in vitro models that incorporate macrophages and allow for easy manipulation of cells will be vital in elucidating the role that this signaling pathway plays in macrophage-regulation of angiogenesis. Looking ahead, we foresee more sophisticated experimental systems uncovering an even greater role for macrophage-derived Wnt ligands in regulating angiogenesis.

## Competing interests

The authors declare no competing interests.

## Authors’ contributions

ACN and CCWH wrote the paper. Both authors read and approved the final manuscript.
